# Editorial: Natural products and immune inflammation: mechanistic understanding based on systems biology

**DOI:** 10.3389/fphar.2025.1642311

**Published:** 2025-06-24

**Authors:** Zhen Ma, Xianyu Li

**Affiliations:** ^1^ Institute of Materia Medica, China Academy of Chinese Medical Sciences, Beijing, China; ^2^ Experiment Research Center, China Academy of Chinese Medical Sciences, Beijing, China

**Keywords:** immune inflammation, herbal medicine, artificial intelligence, natural compounds, omics

Immune inflammatory response is the core of the body’s defense and repair, but its imbalance is the common underlying mechanism factor for major chronic diseases such as cancer, autoimmune diseases, neurodegenerative diseases, and metabolic syndrome. Faced with these complex diseases, modern drug discovery and development often encounters bottlenecks such as single target, drug resistance, and side effects. Natural products, with their structural diversity and multi-target effects, have been empirically validated through millennia of human use and are becoming valuable therapeutic resources for combating immune inflammation related diseases. However, the complex composition and unclear mechanism of action of natural products are like a “black box”, which seriously hinders their scientific evaluation and clinical translation. The theory of systems biology can provide a new perspective, and we have analyzed 21 excellent articles to explain this viewpoint. The rise of systems biology provides a revolutionary key to unlocking this “black box” and gaining a deeper understanding of the precise network of natural products regulating immune inflammation (Zhang et al., 2019; Li and Zhang, 2013).

The traditional linear research paradigm of “one gene, one protein, one drug” is inadequate for analyzing the typical complex system of “multi-component, multi-target, multi-pathway” in natural products. Systems biology adopts a holistic perspective, integrating multidimensional data such as genomics, transcriptomics, proteomics, metabolomics, and microbiology, and employing bioinformatics and computational models for integrated analysis (Kitano, 2002; Hopkins, 2008). It allows us to go beyond the limitations of single molecular events and provide a holistic view of how natural products disrupt the entire biological network, and ultimately reshape immune homeostasis (Li and Zhang, 2013).

Research driven by systems biology has profoundly revealed the immune regulatory mechanisms of numerous traditional Chinese medicine products and prominent natural product compounds. In the articles included in this Research Topic, it is revealed that ephedra sinica polysaccharides (from *Ephedra sinica*can) modulate the intestinal microbiota and anti-inflammatory immunity of bacterial metabolites in rheumatoid arthritis, increase the levels of IL-1 β and IL-6 in restored mouse serum, and inhibit the entry of inflammatory factors from the intestine into the joints. The elevation of HDAC1 and HDAC2 levels induced by intestinal inflammation leads to NF-κ B phosphorylation and activation of TLR4 and MyD88 in synovial tissue of rheumatoid arthritis, which is a key factor contributing to immune imbalance in rheumatoid arthritis. Through integrated genomic and metabolomic analyses, it was found that the potential mechanism of action of ephedra sinica polysaccharides is enriched in taurine and hypotaurine metabolism, porphyrin metabolism, and the enrichment of short-chain fatty acid (SCFA)-producing bacteria. At the same time, metabolites involved in metabolic pathways may help ephedra sinica polysaccharides inhibit intestinal and synovial inflammation to alleviate rheumatoid arthritis. For natural product monomers, gene transcription testing found that Tanshinone IIA’s (from *Salvia miltiorrhiza*) of the ATM/GADD45/ORC pathway alleviates myocardial ischemia-reperfusion injury, while Tanshinone IIA’s of ATM significantly increases the protein expression levels of ATM, GADD45, and ORC1. By integrating transcriptome and protein interaction network analysis, researchers found that curcumin not only directly inhibits the expression of pro-inflammatory factors TNF - α and IL - 6, but also exerts broad-spectrum anti-inflammatory effects by regulating key JAK - STAT, NF - κ B, and MAPK signaling pathways (Aggarwal and Harikumar, 2009; He et al., 2015). The polyphenol EGCG in green tea demonstrates another advantage of systems biology - revealing host microbe interactions. Integrated metagenomic and metabolomic analyses demonstrated that EGCG significantly remodels the structure of intestinal microbiota, enrich beneficial bacteria producing short chain fatty acids, and enhance the expression of proteins critical for intestinal barrier integrity, thereby reducing systemic low-grade inflammation (Yang et al., 2016). The therapeutic evolution of artemisinin (derived from *Artemisia annua*) from antimalarial agents to immunomodulatory agents also benefited from systematic analysis. Research has found that its derivatives can activate the transcription factor Nrf2 pathway, induce the expression of a series of antioxidant enzymes, and inhibit the excessive activation of NLRP3 inflammasomes, demonstrating significant immunomodulatory and joint-protective effects in rheumatoid arthritis models (Wang et al., 2015; Krishna et al., 2008).

However, the journey of systems biology in natural product research still faces multiple challenges. The complexity of Chinese herbal medicine is the primary obstacle. The identification of active ingredients, interactions among each component, and their contributions to *in vivo* metabolic transformation products constitute an extremely complex network system, which has long hindered scientific interpretation and precise application (Gertsch, 2011; Efferth and Koch, 2011). The heterogeneity and dynamics of biological systems are equally crucial. The significant genetic background, epigenetic status, baseline immune status, and gut microbiota differences among individuals result in highly variable responses to the same natural product (Bashiardes et al., 2017; Zmora et al., 2019). The gap between system level insights and clinically effective interventions urgently needs to be bridged. How to transform complex network model predictions into actionable and precise therapeutic strategies, such as patient stratification, dose optimization, and combination therapies, and designing a experimental frameworks capable of capturing these network effects, remains a major challenge in current research (Barabási et al., 2011; He et al., 2023).

The rise of high-throughput omics technology has provided revolutionary tools to solve this problem (Wang et al., 2015). Proteomics can comprehensively depict the dynamic changes in inflammation-related protein expression profiles, post-translational modifications, and signaling pathways following traditional Chinese medicine intervention, accurately targeting key effector proteins and targets (Chen et al., 2020). Combining metabolomics analysis of small molecule metabolite disturbances can reveal the impact of traditional Chinese medicine on inflammation related metabolic reprogramming. Transcriptomics elucidates upstream regulatory mechanisms at the gene expression level. The collaborative application of these omics technologies not only enables the systematic analysis of the mechanism of action network of classical formulas, surpassing the limitations of single-target research (Wu et al., 2024), but also enables the discovery of potential specific combinations of biomarkers such as cytokines, acute phase proteins, metabolites, etc. For efficacy evaluation and disease classification (Chen et al., 2020). This has laid a solid foundation for modernizing traditional Chinese medicine formulas, guiding clinical precision medication, and developing innovative Chinese medicine formulations based on clear targets. In the future, the integration of multi omics data in systems biology and artificial intelligence analysis (Wu et al., 2024) will further promote the research on traditional Chinese medicine immune inflammation regulation from a “black box” to a more transparent and more precise stage, and accelerate its integration into international scientific and medical practice.
